# Inhibition of TRF2 accelerates telomere attrition and DNA damage in naïve CD4 T cells during HCV infection

**DOI:** 10.1038/s41419-018-0897-y

**Published:** 2018-09-05

**Authors:** Lam Nhat Nguyen, Juan Zhao, Dechao Cao, Xindi Dang, Ling Wang, Jianqi Lian, Ying Zhang, Zhansheng Jia, Xiao Y. Wu, Zheng Morrison, Qian Xie, Yingjie Ji, Zheng Zhang, Mohamed El Gazzar, Shunbin Ning, Jonathan P. Moorman, Zhi Q. Yao

**Affiliations:** 1 Center of Excellence in Inflammation, Infectious Disease and Immunity, James H. Quillen College of Medicine, East Tennessee State University, Johnson City, TN 37614 USA; 2 Division of Infectious, Inflammatory and Immunologic Diseases, Department of Internal Medicine, Quillen College of Medicine, ETSU, Johnson City, TN 37614 USA; 3 Department of Infectious Diseases, Tangdu Hospital, The Fourth Military Medical University, Xi’an, China; 4 Research Center for Clinical and Translational Medicine, Beijing 302 Hospital, Beijing, China; 5 Department of Veterans Affairs, Hepatitis (HCV/HBV/HIV) Program, James H. Quillen VA Medical Center, Johnson City, TN 37614 USA

## Abstract

T cells play a crucial role in viral clearance and vaccine responses; however, the mechanisms that regulate their homeostasis during viral infections remain unclear. In this study, we investigated the machineries of T-cell homeostasis and telomeric DNA damage using a human model of hepatitis C virus (HCV) infection. We found that naïve CD4 T cells in chronically HCV-infected patients (HCV T cells) were significantly reduced due to apoptosis compared with age-matched healthy subjects (HSs). These HCV T cells were not only senescent, as demonstrated by overexpression of aging markers and particularly shortened telomeres; but also DNA damaged, as evidenced by increased dysfunctional telomere-induced foci (TIF). Mechanistically, the telomere shelterin protein, in particular telomeric repeat binding factor 2 (TRF2) that functions to protect telomeres from DNA damage, was significantly inhibited posttranscriptionally via the p53-dependent Siah-1a ubiquitination. Importantly, knockdown of TRF2 in healthy T cells resulted in increases in telomeric DNA damage and T-cell apoptosis, whereas overexpression of TRF2 in HCV T cells alleviated telomeric DNA damage and T-cell apoptosis. To the best of our knowledge, this is the first report revealing that inhibition of TRF2 promotes T-cell telomere attrition and telomeric DNA damage that accelerates T-cell senescent and apoptotic programs, which contribute to naïve T-cell loss during viral infection. Thus, restoring the impaired T-cell telomeric shelterin machinery may offer a new strategy to improve immunotherapy and vaccine response against human viral diseases.

## Introduction

T cells play a pivotal role in controlling viral infection and vaccine responses; however, the mechanisms underlying T-cell dysfunction that lead to chronic infection and poor vaccine response remain unclear. Hepatitis C virus (HCV) is highly efficient at establishing chronic infection, thus becoming an excellent model to study the mechanisms of T-cell dysregulation and viral persistence^1^.

Recently, we and others have found that HCV infection can accelerate T-cell aging, as evidenced by overexpression of aging markers and attrition of telomeres, indicating excessive cell proliferative turnover or inadequate telomeric DNA maintenance^2–9^. Telomeres are repeating hexameric DNA sequences that are found at chromosome ends in association with a complex of shelterin proteins. Telomere integrity is a key feature of linear chromosomes that preserve genome stability and function, whereas telomere erosion is a hallmark of cell senescence that drives cell dysfunction or apoptosis^10,11^. Although telomere length is maintained in most cases by the telomerase, shelterin is essential to protect telomeres against unwanted DNA damage response (DDR)^12,13^. Shelterin comprises six polypeptides (TRF1, TRF2, RAP1, TIN2, TPP1, and POT1), of which telomeric repeat binding factor 2 (TRF2) is a key factor that plays an essential role in maintaining telomere integrity^14^. TRF2 also protects chromosome ends against replicative DNA damage, particularly those that occur due to topological stress^15^. Notably, TRF2 expression is increased in a variety of human cancers; consistently, its downregulation reduces tumorigenicity^16,17^. The role of TRF2 in reprogramming telomeric DNA damage and remodeling T-cell homeostasis during viral infection, however, is largely unknown.

To identify factors that perturb T-cell homeostasis during viral infection, we have explored the role of TRF2 in protecting telomeric DNA damage and T-cell apoptosis with a model of HCV infection. We provide evidence revealing that TRF2 inhibition promotes telomere attrition and DNA damage during HCV infection, rendering HCV T cells more senescent and apoptotic, thus potentially contributing to the HCV persistence and vaccine non-responsiveness.

## Materials and methods

### Subjects

The study protocol was approved by the institutional review board (IRB) of East Tennessee State University and James H. Quillen VA Medical Center (ETSU/VA IRB, Johnson City, TN). Written informed consent was obtained from each patient included in this study. The study subjects were composed of two populations: 180 chronically HCV-infected patients and 160 age-matched healthy subjects (HSs). All HCV-infected patients were positive for HCV RNA, prior to antiviral treatment. HSs, obtained from Physicians Plasma Alliance (PPA), Gray, TN, were negative for HBV, HCV, and HIV infection.

### Cell isolation and culture

Peripheral blood mononuclear cells (PBMCs) were isolated from whole blood by Ficoll (GE Healthcare, Piscataway, NJ) density centrifugation. Naïve and memory CD4^+^ T cells were isolated from PBMCs using the Naïve or Memory CD4^+^ T Cell Isolation Kit and a MidiMACS™ Separator (Miltenyi Biotec Inc., Auburn, CA). The isolated T cells were cultured in RPMI-1640 medium containing 10% fetal bovine serum (Atlanta Biologicals, Flowery Branch, GA), 100 IU/ml penicillin and 2 mM l-glutamine (Thermo Scientific, Logan, Utah) at 37 °C and 5% CO_2_ atmosphere.

### Flow cytometry

For phenotypic analysis of T cells, PBMCs were stained with CD3-PE, CD4-APC, CD45RA−FITC, and CD28-PerCP/Cy5 antibodies or isotype controls (BioLegend, San Diego, CA). CD39-PE and CD57-APC (BioLegend) were employed to assess senescent status of CD4 T cells. To determine cell apoptosis, PBMCs were stained with CD45RA−FITC, CD4-APC along with Annexin V (Av)-PE and 7-aminoactinomycin D (7AAD) (BD Biosciences, San Jose, CA) following the manufacturer’s protocol. Reactive oxygen species (ROS) were measured using the 2ʹ,7ʹ-Dichlorofluorescin Diacetate (DCFDA)−based Cellular ROS Detection Kit (Abcam, Cambridge, MA) according to manufacturer’s protocol. Flow cytometric analysis, gating strategy, and background controls were performed as described previously^6^.

### Flow-FISH

Telomere length was measured by Flow-FISH^18^. Briefly, PBMCs were stained with CD4-Alexa-647, and fixed in Cell Fixation buffer (BioLegend) for 20 min. Cells were incubated with telomere probe TelC (5ʹ-CCCTAACCCTAACCCTAA-3ʹ)-FITC (0.3 μg probe/mL, PNA Bio, Newbury Park, CA) at room temperature for 10 min in the dark and then at 82 °C for 10 min. The cells were washed with post-hybridization buffer, followed by flow cytometry buffer, then stained with CD45RA−perCP/Cy5.5, and analyzed by flow cytometry.

### RNA isolation and real-time RT-PCR

Total RNA was extracted from 1 × 10^6^ cells using PureLink RNA Mini Kit (Invitrogen, Carlsbad, CA), and complementary DNA was synthesized using High Capacity cDNA Reverse Transcription Kit (Applied Bio systems, Foster city, CA) per the manufacturer’s instruction. Quantitative real-time PCR was performed in triplicate as described previously^4^. Gene expression was normalized to GAPDH levels and is presented as fold changes using the 2^-ΔΔct^ method. PCR primer sequences are shown in Table 1.

**Table 1:** Primers sequences for quantitative RT-PCR

**Target gene**	**Sequence 5′ to 3′**
hTERT-F	CCAAGTTCCTGCACTGGCTGA
hTERT-R	TTCCCGATGCTGCCTGACC
TERF1-F	TGCTTTCAGTGGCTCTTCTG
TERF1-R	ATGGAACCCAGCAACAAGAC
TERF2-F	GGTACGGGGACTTCAGACAG
TERF2-R	CGCGACAGACACTGCATAAC
POT1-F	TTCCACTAAAGAGCAGGCAA
POT-R	TGAAGTTCTTTAAGCCCCCA
TINF2-F	CCAGAAAGGGTTCCCCATAC
TINF2-R	TTTACCAGCAGGTGAAGCAG
TERF2IP-F	TCTTCTTCAGGCAAATCTGGA
TERF2IP-R	CCTCCTCCCAGAAGCTCAA
TPP1-F	TCACCAGATCAGCCACATTC
TPP1-R	GGAAAGACTCTCGGAGCTG
TP53-F	ATGGAGGAGCCGCAGTCAGAT
TP53-R	GCAGCGCCTCACAACCTCCGTC
CDKN1A-F	CGATGGAACTTCGACTTTGTCA
CDKN1A-R	GCACAAGGGTACAAGACAGTG
CDKN2A-F	AGACTTGGGTGGAAGAGGA
CDKN2A-R	TAATCATCACAGCTGTTCGG
GAPDH-F	TGCACCACCAACTGCTTAGC
GAPDH-R	GGCATGGACTGTGGTCATGAG

### Western blotting

Naïve CD4 T cells (2 × 10^6^) purified from HCV patients and HSs were used for western blot as described previously^6^. Primary and secondary antibodies included TRF2, TPP1, RAP1, TIN2, γH2AX, phospho-p53^ser15^, p21, PARP-1, caspase-3, GAPDH, β-actin (Cell Signaling), POT1 (R&D System, Minneapolis, MN), TRF1 (Thermo Fisher), p53, Ub (Santa Cruz, Dallas, Texas), Siah1 (Abcam, Cambridge, MA), and horseradish peroxide-conjugated antibody (Cell Signaling). Images were captured using ChemiDoc™ XRS + System (Bio-Rad). Protein band intensity was quantitated by Image Lab software (Bio-Rad).

### Co-immunoprecipitation (Co-IP) and ubiquitination assay

Naïve CD4 T cells (6 × 10^6^) were lysed in ubiquitination assay buffer supplemented with 0.1% sodium dodecyl sulfate (SDS). Equal amount of cell lysates were subjected to Co-IP assay by adding TRF2 monoclonal antibody or IgG control, and overnight rotated at 4 °C. Next, 50 μl protein A/G agarose beads (Santa Cruz) were added to each sample, incubated at 4 ^o^C for 1.5 h with rotation. Conjugated beads were washed five times with ice-cold ubiquitination assay buffer, then subjected to SDS-polyacrylamide gel electrophoresis and western blot as described above.

### Confocal microscopy

Naïve CD4 T cells were isolated and cultured as described above, followed by immunofluorescence staining using a method described previously^6^. Rabbit anti-53BP1 (Cell Signaling) and mouse TRF1 (Thermo Fisher) were used as primary antibodies and anti-rabbit IgG-Alexa Fluor 488 and anti-mouse IgG- Alexa Fluor 555 (Invitrogen) were used as secondary antibodies. Then, cells were washed and mounted with DAPI Fluoromount-G (SouthernBiotech, Birmingham, AL). Images were acquired with a confocal laser-scanning inverted microscope (Leica Confocal, Model TCS sp8, Germany).

### siRNA silencing

Naïve CD4 T cells (5 × 10^6^) isolated from HSs were transfected with 100 nM of Smartpools siRNA targeting 4× sequences on TERF2 open-reading frame or scramble siRNA control (Dharmacon, Lafayette, CO), using the Human T Nucelofector Kit and Nucleofector I Device (Lonza, Allendale, NJ) following the manufacturer’s instruction. After 72 h, the cells were harvested and analyzed by flow cytometry, western blotting, and confocal microscopy.

### Lentivirus transduction

For lentiviral packaging, HEK293T cells at 80% confluency were transfected with 2.5 μg of pMD2.G (# 12259), 7.5 μg of psPAX2 (# 12260) (both gifts from Dr. Didier Trone, Addgene), and 10 μg pWPiR or pWPiR-TRF2 plasmids, which contain Internal Ribosome Entry Site (IRES)-driven Green Fluorescent Protein (GFP) protein expression (generous gifts from Dr. Eric Gibson and Dr. Vincent Picco) using Transporter^TM^ 5 (Polyscience, Inc, Warrington, PA) reagent following the manufacturer’s instruction. After 4 days, cells were harvested and subjected to flow cytometry and western blotting.

### Statistics

The data were analyzed using Prism 7 software, and are presented as mean ± SEM or median with interquartile range. Comparisons between two groups were made using unpaired Student’s *t*-test, or paired *Tt*-test. Multiple groups were analyzed by ANOVA, with a Tukey’s test or a nonparametric Mann–Whitney *U*-test. *P*-values < 0.05, < 0.01, or < 0.001 were considered statistically significant or very significant, respectively.

## Results

### T-cell homeostasis and apoptosis in HCV-infected patients versus age-matched HS

**Fig. 1:**
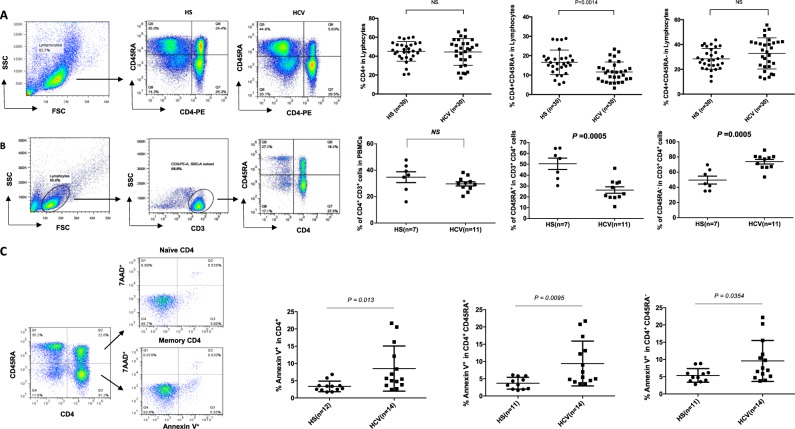
T-cell homeostasis and apoptosis in HCV-infected patients versus age-matched HS. **a** T-cell homeostasis analysis by flow cytometry. Representative dot plots and summary data for percentages of total CD4^+^, CD45RA^+^CD4^+^ naïve, and CD45RA^-^CD4^+^ memory T-cell frequencies within PBMCs from 24 HCV patients and 24 HSs. Each symbol represents one subject. Data are expressed as mean ± SE. NS nonsignificant. **b** T-cell homeostasis analysis by flow cytometry. PBMCs were first gated on lymphocytes, then CD3^+^ T cells, and subsequently CD4^+^CD45RA^+^ naïve and CD4^+^CD45RA^-^ memory T cells from 11 HCV patients and 7 HSs. **c** PBMCs isolated from 14 HCV patients and 11 HSs were analyzed for Av and 7AAD expression by flow cytometry. Representative dot plots and summary data for the percentages of cell apoptosis are shown. Given the data normality distribution, total CD4 Av is shown as median with interquartile range, and its *P-*value was calculated by nonparametric test; naïve and memory T-cell Av is shown as mean ± SE, *P*-value was calculated by *t*-test

Dysregulated T-cell homeostasis is a characteristic of persistent viral infection; however, the mechanisms that control T-cell homeostasis and virus persistence in humans remain unclear^19^. As an initial approach to identify factors that perturb T-cell homeostasis in HCV infection, we first analyzed total CD4^+^, naïve CD4^+^CD45RA^+^, and memory CD4^+^CD45RA^−^ T-cell subsets in PBMCs isolated from HCV and HS. As shown in Fig. 1a, although the percentage of total CD4^+^ T-cell frequencies were similar in HCV patients and HS, the naïve CD4 T-cell repertoire was significantly contracted, whereas memory CD4 T cells expanded, in HCV-infected patients. To exclude the possibility of gating CD4^+^ monocytes, we gated on lymphocytes and then CD3^+^ T cells, followed by analysis of CD3^+^CD4^+^CD45RA^+^ (naïve) and CD3^+^CD4^+^CD45RA^−^ (memory) T-cell subsets. This analysis yielded the same results (Fig. 1b). In addition to analyzing CD4 T-cell frequencies by flow cytometry, we also examined the absolute CD4 T-cell numbers by purifying naïve CD4 T cells from peripheral blood of HCV patients versus HS. Base on the isolated PBMCs and the yield of naïve CD4^+^ T cells, we observed a significantly lower number of naïve CD4 T cells in the blood of HCV patients compared with HS (Table S1). These findings of contracted naïve CD4 and expanded memory CD4 T-cell subsets are consistent with previous reports by us and others^6,20^ showing reduced naïve CD4 T cells, suggesting a state of cell activation/differentiation followed by exhaustion/senescence in patients with chronic HCV infection.

The total size of the T-cell repertoire is well maintained by a fine balance between influx of newly generated T cells from the thymus, efflux by consumption of programmed cell death, and self-replication within the existing pool of lymphocytes^21^. With deficient influx from the thymus in adults, the immune system reacts by expanding existing T cells, leading to increased proliferative turnover, cell senescence, and ultimately, cell apoptosis^21^. To explore the contribution of apoptosis to T-cell homeostasis during HCV infection, PBMCs derived from HCV patients were compared with HS for the expression of Av and 7AAD. As shown in Fig. 1c, Av expression in CD4 T cells revealed an increased rate of apoptosis in HCV patients in total CD4, as well as naïve and memory CD4 T cells. Notably, naïve CD4 T cells were more apoptotic than memory CD4 T cells in HCV patients. This apoptotic susceptibility of T cells in HCV patients may necessitate compensatory homeostatic proliferation that can lead to telomere attrition and cell senescence.

### T-cell premature senescence in HCV-infected patients versus age-matched HS

**Fig. 2:**
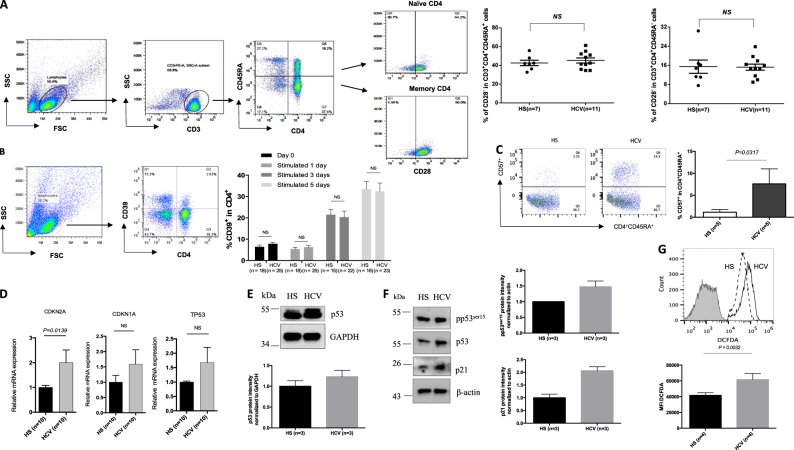
T-cell senescence in HCV-infected patients versus age-matched HS. **a** CD28 expression on CD3^+^CD4^+^CD45RA^+^ naïve and CD3^+^CD4^+^CD45RA^-^ memory T cells from 11 HCV-infected patients and 7 age-matched HSs. The gating strategy and summary data of the flow cytometry analysis are shown. Each symbol represents one subject. NS non significant. **b** CD39 expression in T cells from HCV patients and HS. PBMCs isolated from HCV subjects and HSs were cultured in the presence of anti-CD3/CD28 for 0, 1, 3, 5 days, followed by flow cytometric analysis for CD39 expression on CD4^+^ T cells. Representative dot plots and summary data are shown. **c** CD57 expression in naïve T cells from HCV patients and HS. PBMCs were stimulated with anti-CD3/CD28 for 3 days and then analyzed for CD57 expression on CD4^+^CD45RA^+^ naïve T cells isolated from five HCV patients and five HSs. **d** mRNA expression of CDKN2A (p16), CDKN1A (p21), and TP53 in naïve CD4 T cells isolated from 10 HCV patients and 10 HSs. The purified CD4^+^CD45RO^−^ naïve T cells were analyzed by real-time RT-PCR for p16^ink4a^, p21^cip1^, and TP53 mRNA expression. Values were normalized to GAPDH expression and are presented relative to HS. **e** Western blot analysis for p53 expression in naïve CD4 T cells isolated from HCV patient and HS. GAPDH serves as a loading control. Representative WB imaging and summary densitometry data are shown. **f** Western blot analysis for pp53, p53, p21 expressions in TCR-stimulated naïve CD4 T cells isolated from HCV patient and HS. β-Actin is used as loading control. Representative WB imaging and summary densitometry data are shown. **g** Measurement of ROS level in naïve CD4 T cells. Naïve CD4 T cells were isolated from HCV patients and HS and subjected to ROS measurement using the DCFDA-based Cellular ROS Detection Kit. Representative overlaid histogram and summary data for the MFI of DCFDA level in naïve CD4 T cells from HCV patients and HS are shown (*n* = 4)

To determine the role of homeostatic proliferation in T-cell senescence, we assessed the aging markers in T cells from HCV patients and HS. Because loss of CD28 (a T-cell receptor (TCR) co-stimulatory molecule required for T-cell activation and survival) is considered an unequivocal marker for T-cell senescence^22^, we first measured CD28 expression on CD3^+^CD4^+^CD45RA^+^ naïve T cells and CD3^+^CD4^+^CD45RA^-^ memory T cells. As shown in Fig. 2a, we did not observe any difference in CD28 expression on T-cell subsets between HCV patients and HS. We also measured the expression of CD39 (a cell surface-located ATPase that identifies terminally differentiated cells^23^) on CD4 T cells following anti-CD3/CD28 stimulation for 0, 1, 3, and 5 days. Again, no differences were detected in CD39 expression on either the resting or TCR-stimulated CD4 T cells in HCV patients versus HS (Fig. 2b). We next measured the expression levels of CD57 (also known as human natural killer 1, HNK1), a glycoprotein expressed on senescent NK or T lymphocytes^24^. Remarkably, at day 3 of TCR stimulation, CD57 expression on CD4^+^CD45RA^+^ naïve T cells was significantly increased in HCV patients compared with HS (Fig. 2c).

To better define the aging process in naïve T cells during viral infection, we assessed the aging-associated cell cycle inhibitors^25^ including p16^ink4a^, p21^cip1^, and p53. As shown in Fig. 2d, HCV naïve T cells exhibited higher mRNA levels of CDKN1A (encodes p21^cip1^), CDKN2A (encodes p16^ink4a^), and p53, as determined by real-time RT-PCR. Although we could not detect p16^ink4a^, p21^cip1^, and pp53^ser15^ protein expressions in resting naïve CD4 T cells by western blot, we observed increases in total p53 protein expression in HCV T cells compared with HS (Fig. 2e). In addition, we found increases in pp53^ser15^, total p53, and p21^cip1^ protein expressions in TCR-stimulated naïve CD4 T cells from HCV patients (Fig. 2f). We also examined the expression of aging makers in memory CD4 T cells at both mRNA and protein levels. As shown in supplemental Fig. S1, we did not observe any difference in mRNA expression of TP53, CDKN1A, and CDKN2A (Fig. S1A). However, we observed increases in p53 protein expression and cleaved poly ADP-ribose polymerase 1 (PARP-1) in unstimulated memory CD4 T cells (Fig. S1B), as well as increases in p21 and γH2AX levels in TCR-stimulated memory CD4 T cells (Fig. S1C) derived from HCV patients, suggesting that memory CD4 T cells also overexpress aging proteins during HCV infection. These findings reaffirm our previous observations that aging markers are upregulated in CD4 T cells during chronic HCV infection^3–5,26^.

Along with others, we have shown that naïve CD4 T cells are typically resistant to Fas/Fas-L-mediated apoptosis, pointing to cell internal signals as apoptosis initiators^27^. One of the internal stressors linked to cell apoptosis is damaged DNA, which is particularly important in senescent cells chronically exposed to endogenously generated ROS^28^. To determine whether ROS can cause DNA damage and cell apoptosis during viral infection, we measured the ROS levels in naïve CD4 T cells isolated from HCV and HS by flow cytometry using the DCFDA, a fluorogenic dye that measures ROS within the cells^29^. Indeed, the mean fluorescence intensity (MFI) of DCFDA was significantly increased in naïve (Fig. 2g), as well as memory (Fig. S1D) CD4 T cells derived from HCV patients compared with HS, indicating that ROS generated during infection may play a role in DNA damage and cell apoptosis.

### Telomere attrition and DNA damage in T cells from HCV patients versus age-matched HS

**Fig. 3:**
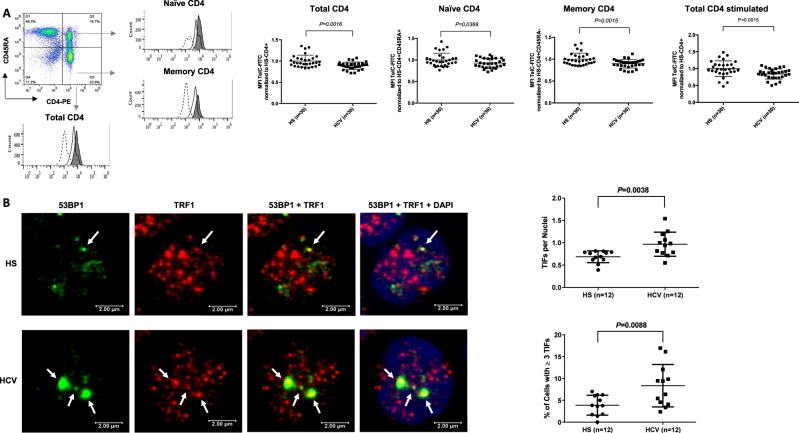
Telomere length and telomeric DNA damage in T cells from HCV patients and HS. **a** Telomere attrition in CD4 T cells from HCV patients. PBMCs were isolated from 24 HCV-infected patients and 24 age-matched HSs (with or without TCR stimulation), followed by Flow-FISH analysis for telomere length in total CD4, naïve and memory CD4 T cells. The gating strategy, overlaid histograms (dashed lane = isotype control; solid lane = HCV patient; filled area = HS) and summary data of mean fluorescence intensity (MFI) are shown. Each symbol represents one subject. The relative MFI of HCV telomere length was normalized by HS. **b** Telomeric DNA damage in naïve CD4 T cells derived from chronically HCV-infected individuals versus HS was analyzed by confocal microscopy for colocalization of 53BP1 and TRF1. Representative imaging and summary data for the numbers of TIFs per nuclear, as well as the percentage of cells with > 3 TIFs are shown (*n* = 12)

As telomere attrition is a hallmark of cell senescence, we further characterized T-cell senescence in HCV infection by determining telomere length in total, naïve and memory CD4 T cells by Flow-FISH. As shown in Fig. 3a, telomere length was significantly shortened in HCV-derived total CD4 T cells, as well as in naïve and memory CD4 T cells compared with HS. In addition, telomere loss was observed in activated CD4 T cells following TCR stimulation for 3 days.

As mammalian telomeres consist of plentiful triple guanine repeats (TTAGGG) that are very sensitive to oxidative DNA damage, we speculate that telomeres in HCV T cells are not only shortened, but more importantly, DNA damaged. Notably, following genotoxic insult, 53BP1 is recruited to the DNA damage site and acts as a docking station for other adaptor proteins to form microscopically visible nuclear foci (DNA damage foci)^30^. Thus, identifying dysfunctional telomere-induced foci (TIF) is typically regarded as a hallmark of telomeric DDR^30^. To determine telomeric DNA damage in T cells during HCV infection, we compared the number of TIFs per nucleus and the percentages of cells with > 3 TIFs by examining the colocalization of 53BP1/TRF1 using confocal microscopy^31,32^. As shown in Fig. 3b, the number of TIF per nucleus, as well as the percentage of T cells with > 3 TIFs were significantly higher in CD4 T cells derived from HCV patients compared with HS. These results suggest that telomeres in patients with chronic HCV infection are not only shortened but also sustain DNA damage, which may cause cell apoptosis, emphasizing the role of telomere integrity in securing T-cell survival.

### Telomeric shelterin proteins in T cells from HCV-infected patients versus age-matched HS

**Fig. 4:**
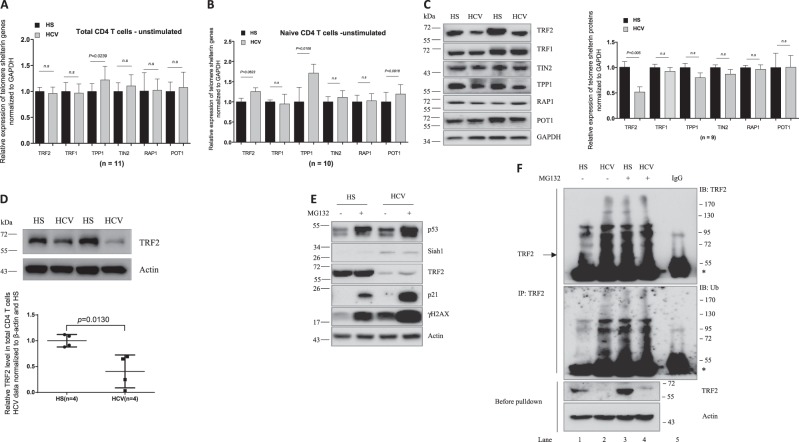
Telomere shelterin mRNA and protein levels in T cells from HCV patients and HS. **a**, **b** mRNA expression of telomere shelterin proteins in total CD4 T cells and naïve CD4 T cells. Total CD4 T cells and CD4^+^CD45RO^−^ naïve CD4 T cells were isolated from 10 HCV patients and 10 HSs. Total RNA was isolated and analyzed by real-time RT-PCR for shelterin mRNA expression. Values were normalized to GAPDH mRNA and calculated relative to HS. **c** Shelterin protein expressions in naïve CD4 T cells isolated from HCV patients and HS. GAPDH is used as loading control. Representative imaging and summary data for western blot densitometry are shown (*n* = 9). **d** TRF2 level in total CD4 T cells from HCV patients and HSs. Representative imaging and summary data for western blot are shown (*n* = 4). The HCV densitometry data were first normalized to β-actin and then HS. **e** Naïve CD4 T cells isolated from HCV and HSs were cultured for 72 h in the presence of DMSO control or proteasome inhibitor MG132 (10 μM) for the last 4 h, followed by western blot analysis for p53, Siah-1, TRF2, p21, γH2AX expressions. β-Actin serves as loading control. **f** Proteasomal degradation of TRF2 through ubiquitin signaling pathway in T lymphocytes during HCV infection. Naïve CD4 T cells isolated from HCV (lanes 2 and 4) and HS (lanes 1 and 3) were lysed in immunoprecipitation (IP) buffer with 0.1% SDS. Protein concentrations were equalized and small amount of cell lysates were saved before the pull-down assay (bottom panel) and used as control. The rest of cell lysates were used for IP with TRF2 antibody or IgG control (lane 5). Immunoprecipitated complexes were pulled-down by protein A/G bead and subjected to immunoblotting with the indicated antibodies

To determine the cause of telomere attrition in T cells during HCV infection, we next investigated the integrity of telomeric shelterin proteins that function to protect telomeres from unwanted DNA damage^12^. We first examined their mRNA expression, by real-time RT-PCR, in T cells isolated from HCV patients and HS. As shown in Fig. 4a, there were no significant difference in their mRNA levels, except TPP1 that was upregulated in total CD4 T cells from HCV-infected patients. Notably, TPP1 was also significantly upregulated, whereas TRF2, TIN2, and POT1 slightly elevated, in HCV-derived naïve CD4 T cells without stimulation (Fig. 4b). As changes in mRNA expression may not necessarily be linear to their protein levels within the cells, we also examined their protein levels in naïve CD4 T cells isolated from HCV patients and HS by western blot. In contrast to their mRNA transcripts, the TRF2 protein level was significantly downregulated, whereas the TRF1, TPP1, and TIN2 proteins were slightly decreased in HCV T cells compared with HS (Fig. 4c). Notably, TRF2 protein inhibition was also observed in total CD4 T cells isolated from HCV patients compared with HS (Fig. 4d). These results indicate TRF2 inhibition, at the posttranscriptional level, in CD4 T cells during HCV infection.

### Mechanisms involved in inhibiting TRF2 protein expression in T cells during HCV infection

As p53-mediated proteasomal degradation via E3 ubiquitin ligase Siah-1a is a major mechanism for regulating TRF2 protein stability in fibroblasts^33^, we examined these proteins by western blot in HCV- and HS-derived naïve CD4 T cells treated with or without the proteasomal inhibitor MG132. As shown in Fig. 4e, p53 protein expression was significantly increased in naïve CD4 T cells from HCV patients versus HS, with or without MG132 treatment. Along with p53 upregulation, the expression of E3 ubiquitin ligase Siah-1a was also increased in both MG132-treated and untreated naïve CD4 T cells derived from HCV patients compared with HS, which was accompanied by decreases in TRF2 expression. In addition, expression of p21^cip1^, a p53 downstream cell cycle inhibitor and cell senescence marker, was significantly elevated after the MG132 treatment, especially in HCV-derived naïve CD4 T cells. Concurrently, the DNA damage marker γH2AX was also markedly elevated in HCV T cells compared with HS, regardless of the MG132 treatment. Collectively, these results suggest that TRF2 inhibition is associated with increases in the p53/Siah-1a signaling in senescent, DNA damaged T cells during HCV infection.

To further address the possibility of ubiquitin degradation as a mechanism for TRF2 inhibition in T cells during HCV infection, we examined the ubiquitination of TRF2 in naïve CD4 T cells with or without MG132 treatment. We performed immunoprecipitation (IP) using a TRF2 monoclonal antibody and then probed the immunoprecipitates with both TRF2- and ubiquitin-specific antibodies. As shown in Fig. 4f, without MG132 treatment, ubiquitinlated TRF2 was significantly higher in HCV T cells, consistent with the lower level of TRF2 before the protein pull-down, compared with HS (lane 2 versus lane 1). These data suggest that ubiquitination-mediated proteasomal degradation of TRF2 occurs in HCV T cells. We also noticed an increased TRF2 ubiquitination in healthy T cells (lane 3 versus lane 1) by the MG132 treatment, but this TRF2 ubiquitination did not increase further in HCV-derived cells (lane 4 versus lane 2), suggesting a highly active TRF2 ubiquitination machinery in HCV T cells. We found a similar pattern of ubiquitin probe in naïve CD4 T cells derived from HCV and HS, with or without MG132 treatment. Taken together, these data indicate an increased proteasomal degradation of TRF2 that is associated with the activation of the p53/Siah-1a ubiquitination pathway in T cells during HCV infection.

### TRF2 plays a key role in protecting telomeres from DNA damage and T-cell apoptosis

**Fig. 5:**
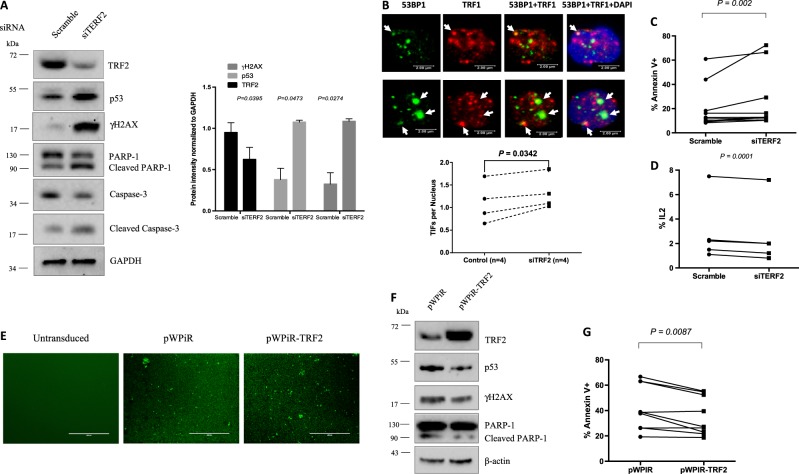
TRF2 plays a key role in protecting telomere from DNA damage and T-cell apoptosis. **a** Representative imaging and summary data of the western blot analysis for TRF2, p53, γH2AX, PARP-1, and caspase-3 expressions in healthy naïve CD4 T cells transfected with TRF2 siRNA (siTERF2) and scrambled siRNA control. GAPDH is shown as a loading control. The experiments were performed using naïve CD4 T cells derived from HS (*n* = 4). **b** Telomeric DNA damage in naïve healthy CD4 T cells. Cells were transfected with control or TRF2 siRNAs and analyzed by confocal microscopy for colocalization of 53BP1 and TRF1. Representative imaging and summary data for numbers of TIFs per nuclear, as well as percentage of cells with > 3 TIFs are shown (*n* = 4). **c** Summary data for apoptotic death of healthy naïve CD4 T cells transfected with the control and TRF2 siRNA (*n* = 10). **d** IL-2 expression in healthy naïve CD4 T cells transfected with the control and TRF2 siRNA. *n* = 5. **e** GFP expression in lentivirus-untransduced and transduced naïve CD4 T cells derived from HCV-infected patients. **f** Representative western blots showing TRF2, p53, γH2AX, and PARP-1 expressions in HCV-derived naïve CD4 T cells transduced with control or TRF2-expressing lentivirus. **g** Percentage of Av^+^ cells in HCV T cells transduced with control or TRF2-expressing lentivirus are shown (*n* = 9)

TRF2 is a key factor in telomere protection and chromosomal stability, which are critical for cell survival and function^14,15^, but its role in T-cell biology in the setting of viral infection remains unknown. To elucidate the role of TRF2 in protecting telomere integrity and T-cell survival, we knocked down TRF2 in healthy T cells and measured DNA damage, cell apoptosis, and cell function. As shown in Fig. 5a, healthy naïve CD4 T cells transfected with siRNA specific to TRF2 (siTERF2) exhibited a significant decrease in TRF2 protein expression compared with cells treated with scramble siRNA. Concurrently, p53 and γH2AX expressions were remarkably upregulated in the TRF2 knockdown T cells. Notably, the expression of caspase-3-dependent cleavage of PARP-1 (an enzyme that catalyzes the transfer of ADP-ribose onto target proteins and plays an important role in maintaining DNA chromosomal stability^34^) was decreased, whereas its cleaved form was increased in T cells after TRF2 knockdown. Correspondingly, caspase-3 was decreased but its cleaved form was increased, which is in line with the increases in apoptosis in TRF2 siRNA-treated cells that have increased p53-mediated DNA damage. Additionally, the numbers of dysfunctional TIF per nucleus (Fig. 5b) and the percentages of Av^+^ apoptotic cells were also significantly increased (Fig. 5c), and the IL-2 expression was substantially decreased in T cells after TRF2 knockdown (Fig. 5d).

To determine whether reconstitution of TRF2 in T cells can alleviate the DNA damage and cell apoptosis occurring during HCV infection, we overexpressed TRF2 in naïve CD4 T cells derived from HCV patients using a lentiviral expression system. As shown in Fig. 5e, lentivirus-mediated, IRES-driven GFP protein expression was observed in T-cell transduced with the pWPiR control vector and the pWPiR-TRF2 construct, but not in untransduced cells. Importantly, TRF2 expression was markedly increased in pWPiR-TRF2-transduced T cells and was accompanied by a decrease in p53, γH2AX, and cleaved PARP-1 levels, indicating an alleviation of p53-mediated DNA damage. In addition, Av expression was significantly reduced in HCV-derived T cells transduced by the pWPIR-TRF2. These data demonstrate that telomere uncapping and recapping is critical for DNA damage and apoptosis, determining T-cell survival and function.

## Discussion

**Fig. 6:**
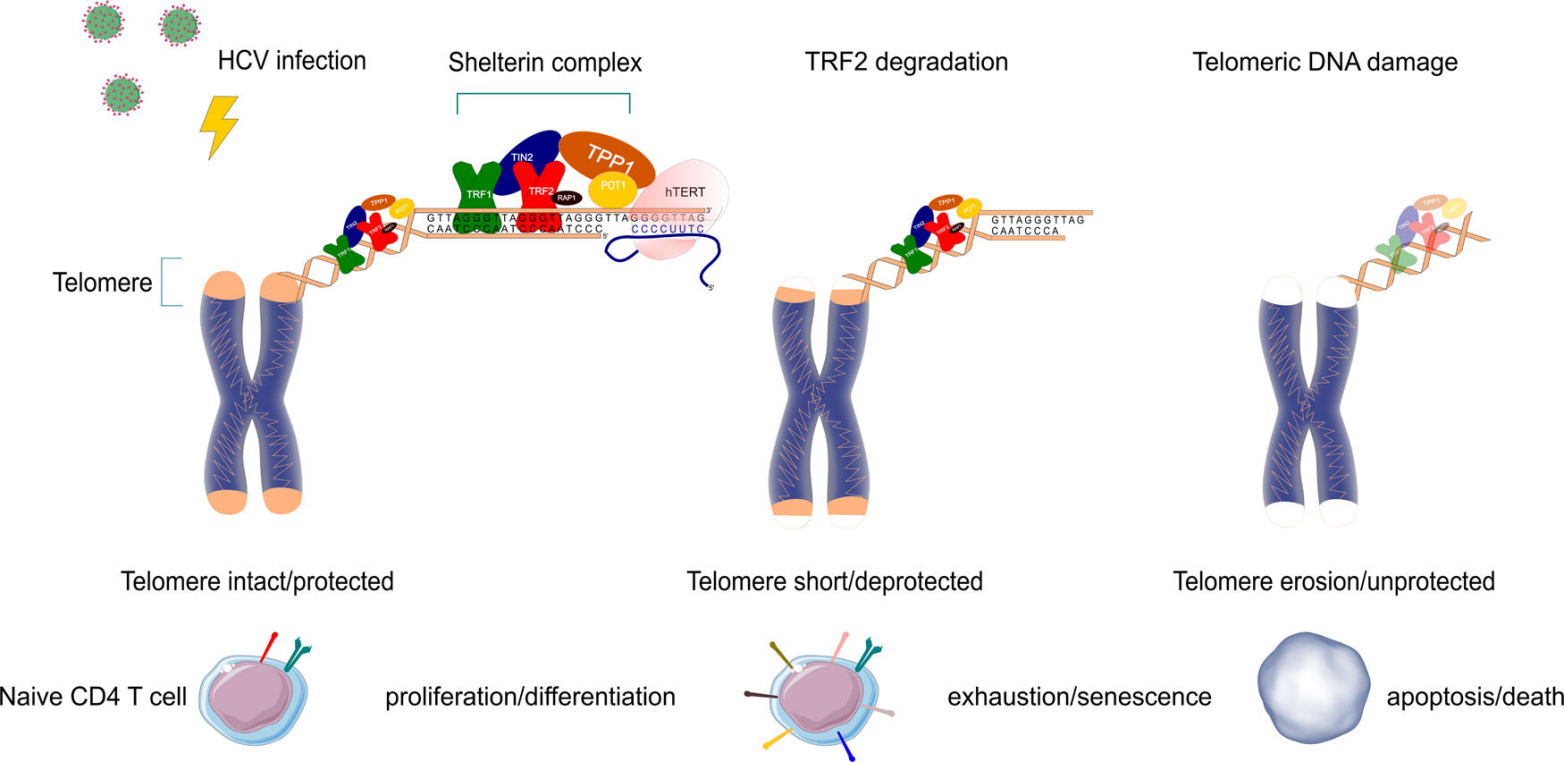
A model of HCV-induced TRF2 inhibition, leading to T-cell telomere uncapping and attrition, accelerating cell senescence, apoptosis, and naïve T-cell loss during HCV infection. HCV infection promotes T-cell activation/proliferation/differentiation and induces TRF2 protein inhibition, which leads to telomere deprotection and triggers DNA damage and telomere erosion, thus contributing to T-cell apoptotic death. Excessive T-cell apoptosis feedback necessitates homeostatic over-expansion, then cell exhaustion/senescence, imposing replicative stress on unprimed naïve T cells and further accelerating naïve T-cell loss. This incessant regulatory cascade represents a novel molecular mechanism underlying T-cell senescence and naïve T-cell loss, which contributes to the viral persistence and vaccine non-responsiveness in human viral infection

Chronic viral infections are characterized by dysfunctional T cells. Here, we show that homeostatic remodeling of the T-cell repertoire during HCV infection primarily affects the naïve T-cell compartment. Specifically, we find that naïve CD4 T cells in HCV patients are senescent, as demonstrated by the overexpression of aging markers, along with telomere attrition with telomeric DNA damage due to loss of the TRF2 protection, thus contributing to increased cell apoptosis. Healthy naïve T cells entered crisis prematurely upon knockdown of TRF2, as evidenced by increased p53 and γH2AX expression, accompanied by increases in the cleaved form of PARP-1 and caspase-3. Accordingly, TRF2 silenced T cells exhibited increased numbers of TIF and apoptosis, concomitant with decreased IL-2 production. In contrast, overexpression of TRF2 in HCV T cells reduced telomeric DNA damage and cell apoptosis. We thus conclude that TRF2 protein inhibition leads to telomere attrition and telomeric DNA damage that triggers cell apoptosis during HCV infection. Based on these novel findings, we propose a model, as depicted in Fig. 6, where HCV-induced telomere deprotection by TRF2 protein inhibition causes accumulation of telomeric DNA damage and telomere erosion, thus contributing to T-cell apoptosis. Telomere attrition-mediated T-cell apoptosis may necessitate homeostatic proliferation and impose replicative stress on unprimed naïve T cells, further contributing to naïve T-cell loss. This represents a novel molecular mechanism that underlies T-cell senescence and T-cell loss.

The immune system is in constant turnover during viral infection, with high demands for lymphocyte replenishment to maintain the T-cell equilibrium. Ongoing antigenic stimulation during chronic viral infection induces continuous differentiation of naïve T cells and turnover of antigen-reactive T cells. In this regard, memory T cells would expand and thus compromise the size and survival of naïve T-cell repertoire. Our study focused on the naïve T cells because they represent the reserves of the immune system, and their survival critically affects the outcome of immune aging. Undoubtedly, the apoptotic loss of naïve T cells determines the generation of sufficient antigen-specific T-cell clones, as well as the cellular yield of homeostatic proliferation, a process that generates new T cells upon the response to neo-antigens, including vaccines. Indeed, along with others, we have shown poor vaccine (HAV, HBV, influenza, and Pneumovax) responses in the setting of chronic viral (HCV and HIV) infections^2,3,35–39^. Our new findings in this study indicate that naïve helper T cells in HCV patients have aberrant abnormalities that jeopardize their ability to mount effective immune (vaccine) responses. In particular, the naïve T cells compartment is severely contracted, and naïve CD4 T cells exhibit telomere attrition with damaged DNA due to the lack of TRF2 protection. Accumulated DNA damage renders HCV T cells more prone to apoptotic death, thus imposing replicative stress and premature senescence.

A typical feature of T cells in chronic viral infection is premature aging, characterized by telomere shortening compared with age-matched HS^2–9^. In normal primary T cells, telomeres undergo shortening at a rate of 50–100 base pairs (bp) per cell division, and predictable loss of telomeric DNA with each cell replication allows telomeres to serve as molecular clock that controls the replicative capacity of T cells before entering cell cycle arrest, senescence, or apoptosis^40,41^. However, telomere loss can increase up to 250 bp per cell cycle during chronic viral infection and, in compensating for this, cell cycle arrest occurs when progressive telomere loss reaches a critical point, a phenomenon known as replicative senescence^40,41^. Based on our studies, we believe that HCV-induced naïve CD4 T-cell loss is primarily driven by ROS-mediated DNA damage and telomere attrition. As depicted in Fig. 6, in healthy young subjects the telomeres are intact with shelterin complexes well formed to protect telomeres from unwanted DNA damage; thus, normal T cells can proliferate/differentiate efficiently in response to antigen stimulation. However, HCV infection can induce TRF2 protein degradation via the p53/Siah1-mediated ubiquitination, leading to telomere uncapping and telomeric DNA damage. We believe that HCV-induced TRF2 inhibition, telomere loss, cellular senescence, and apoptosis are sequential but constant events, that is, if telomeres are mildly or moderately shortening, the over-expanded cells are exhausted or senescent, and cell cycles are arrested in the G1 phase to allow for DNA damage repair. If telomeres are severely shortening to a critical point that the damaged DNAs are irreparable, then the cells will undergo suicidal apoptosis and die. This continuous depletion of naïve CD4 T cells serves as a mechanism and contributes to the high rates of HCV persistence and vaccine non-responsiveness in virally infected individuals.

Several mechanisms may potentially contribute to the senescence-associated telomere attrition. First, increased T-cell proliferative turnover can cause cell division-induced telomere erosion. Typically, telomere is lost due to incomplete synthesis of the terminal DNA during cell division. The enzyme telomerase counteracts telomere loss by synthesizing telomeric repeats during cell proliferation. However, we have found that telomerase expression and its activity in CD4 T cells were unchanged or not dramatically suppressed during HCV infection (unpublished observations), suggesting that other mechanisms might be involved in telomere attrition. In our study, we found a significant decrease in TRF2 and a slight decrease in TRF1, TPP1, and TIN2 protein expressions in HCV T cells. Given that TPP1 and TIN2 are required to bridge the TRF1 and TRF2 complexes for telomere sheltering^42^, we speculate that the relative lower levels of TRF2, TRF1, TPP1, and TIN2 in T cells of HCV patients could lead to poor sheltering, telomere uncapping or deprotection, and thus ROS-mediated DNA damage. Additionally, Zhong et al. reported that the oligonucleotide binding (OB)-fold domain of TPP1 recruits telomerase to telomeres through an association with Telomerase reverse transcriptase (TERT)^43^. Reconstitution of shelterin complexes reveals unexpected stoichiometry that can enhance telomerase processivity^44^. We thus believe that with decreased shelterin proteins, there would be insufficient recruitment of telomerase to telomeres for chromosomal end maintenance during HCV infection. This possibility is under active investigation in our lab.

Second, telomeres are highly susceptible to DNA damage. Human naïve T cells have a relatively long life span (150–160 days) and are exposed to a multitude of genotoxic stressors, causing 1% of approximately 300 billion T cells to be replaced daily. Their telomeric DNA is particularly vulnerable to ROS-induced DDR, even more so than non-telomeric DNA. Plasmid-inserted human telomeres, for example, accumulate sevenfold higher strand breakage than control sequences^45^. In addition, the frequency of single-strand breaks is several-fold higher in telomeres than in the bulk genome when cells are exposed to oxidative stress^46^. In line with these findings, we find that T cells derived from HCV patient exhibit not only shortened telomeres, but also damaged DNA, which can contribute to telomere loss.

Third, an inhibition of protective shelterin proteins may lead to deprotection of the telomeres. In our study, the expression of TRF2 is significantly inhibited at the protein level via the p53/Siah-1a-mediated ubiquitin pathway in naïve CD4 T cells during HCV infection, rendering the uncapped telomeres prone to DNA damage and cell apoptosis. T-cell replicative senescence is induced by uncapped telomeres, which activates DDR and telomere erosion. T-cell death, however, requires overriding of senescence through further telomere attrition, concomitant with loss of DNA damage checkpoints, thus causing cell apoptosis. Notably, TRF2 is a key factor that plays an essential role in maintaining telomere integrity by suppressing the ATM-dependent DDR^14^. Recently, we have shown that telomere loss in HCV T cells is triggered by DDR and the inability of a timely repair by the ATM pathway^6^. Similar to our study, Guo et al.^47,48^ reported that ATM activation in response to ROS was independent of the Mre11-Rad50-Nbs1 complex (MRN) complex. ROS-mediated ATM signaling represses mammalian target of rapamycin complex 1 (mTORC1) signaling and therefore cell growth and proliferation through activation of Tuberous sclerosis complex 2 (TSC2) (a negative regulator of mTOR) by liver kinase B1 and AMP-dependent kinases^49^. ATM engagement of the TSC2/mTORC1 signaling pathway can also regulate autophagy^50^, and differential localization of ATM is correlated with activation of distinct downstream pathways^51^. We have also discovered that KML001, a telomere-targeting drug, can induce telomeric DNA damage and T-cell apoptosis by inhibiting TRF2 expression and impairing the ATM pathway (unpublished observations). Moreover, inhibition of Topoisomerase I or II by camptothecin or ICRF-193, which induces topological stress by suppressing telomere TRF2 protection, also exacerbates telomeric DNA damage and enhances T-cell death (unpublished observations). In this study, we show that TRF2 silencing amplifies telomere uncapping, triggers telomeric DNA damage, and decides cellular fate, suggesting that telomere deprotection via TRF2 inhibition is the underlying molecular mechanism that causes telomeric DNA damage and cell apoptosis in HCV infection. It should be pointed out, however, that the pathogenesis of HCV persistence is multifaceted, in that not only telomere shelterin proteins (especially TRF2) are inhibited, but other mechanisms appear impaired, including DNA repair enzymes (ATM/ATR), telomerase access to telomeres, and DNA topoisomerases, all of which can work in concert to damage telomeres and lead to naïve T-cell loss during HCV infection.

Our findings of TRF2-mediated telomere uncapping and T-cell apoptosis during HCV infection are clinically relevant and highly significant. We propose that TRF2 inhibition is the molecular mechanism that controls T-cell life span in the setting of chronic HCV infection. In line with this, we have recently found that CD4 T cells in latently HIV-infected individuals are also senescent with shortened, DNA-damaged telomeres due to TRF2 and ATM inhibition (unpublished observations). Thus, TRF2-mediated telomere attrition and cell apoptosis may represent a universal mechanism that controls T-cell homeostasis in chronic viral infections. Importantly, our results show that reconstitution of TRF2 is necessary and sufficient to protect telomeres from unwanted DNA damage and rescue HCV T cells from apoptosis, indicating the importance of TRF2 in telomere protection and T-cell survival in human infectious diseases.

These findings might also offer a clinical opportunity for oncogenesis and anticancer treatment, as cell cycle arrest has been associated with tumorigenesis in checkpoint-compromised cells^52^, whereas exacerbation of TRF2-mediated telomere deprotection sensitizes cancer cells to telomere-targeting drugs. Similarly, bone marrow failure and related diseases are often observed in individuals with telomeropathies, which could potentially be explained by cell mitotic arrest resulting from excessive shortened and/or damaged telomeres^53,54^. Therefore, TRF2-mediated telomere uncapping and telomere loss-driven cell cycle arrest may have broader implications through impairing diverse cellular functions.

Telomere integrity is essential to life, as telomere clearly preserves genomic DNA stability and cell proliferative potential. Thus, recognizing telomeric DNA damage due to TRF2 uncapping as a fundamental mechanism of cellular aging will create a new paradigm in cell aging research. Notably, chronic infection or inflammation-induced immunosenescence (i.e., inflammaging) may prove to be a key molecular process that applies to a wide range of clinical scenarios. Nevertheless, to the best of our knowledge, this is the first report showing that inhibition of shelterin protein TRF2 promotes T-cell telomere attrition and telomeric DNA damage to accelerate T-cell senescence and apoptosis in human viral infection. It should be noted that while telomere TRF2 uncapping explains both telomeric DNA damage and cell apoptosis, it may function as a double-edged sword, resulting in both an overwhelming cell death in acute infection and immune tolerance or immune suppression in chronic infection. Our findings indicate that appropriate manipulation of telomere/TRF2 shelterin machinery may restore T-cell competency and prevent premature immune senescence, thus providing a new strategy to improve immunotherapy and vaccine responses against human viral diseases.

## Electronic supplementary material

Supplemental data

Supplementary table 1
