# Supplementary material for: Inhibition of TRF2 accelerates telomere attrition and DNA damage in naïve CD4 T cells during HCV infection
Source: Cell Death Dis. 2018 Sep 5;9(9):900. doi: 10.1038/s41419-018-0897-y (PMC6125360; doi:10.1038/s41419-018-0897-y)
Supplement: Supplementary file 1 — Supplemental data [file 41419_2018_897_MOESM1_ESM.pptx]

## Slide 1
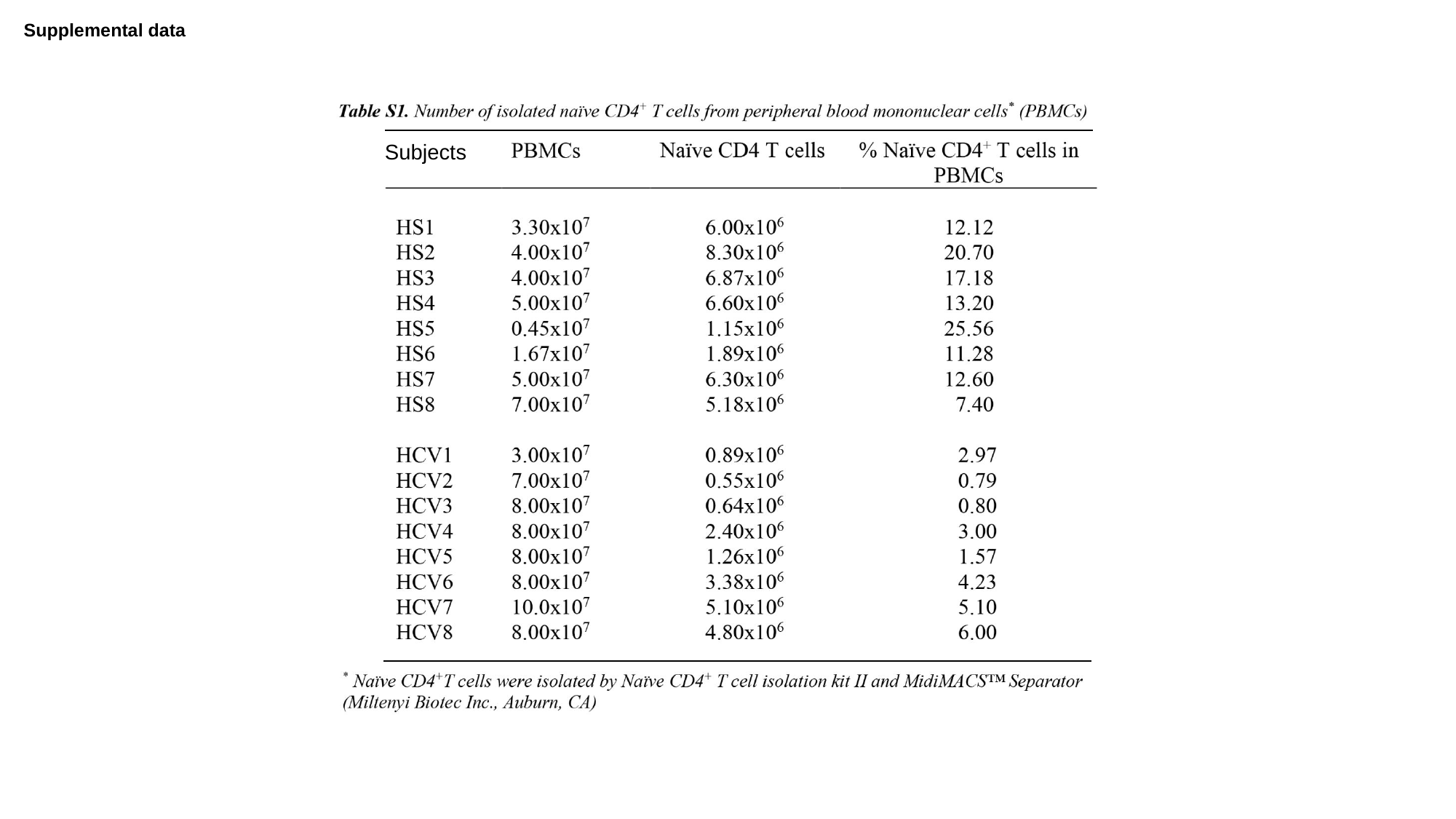

Supplemental data
Subjects

## Slide 2
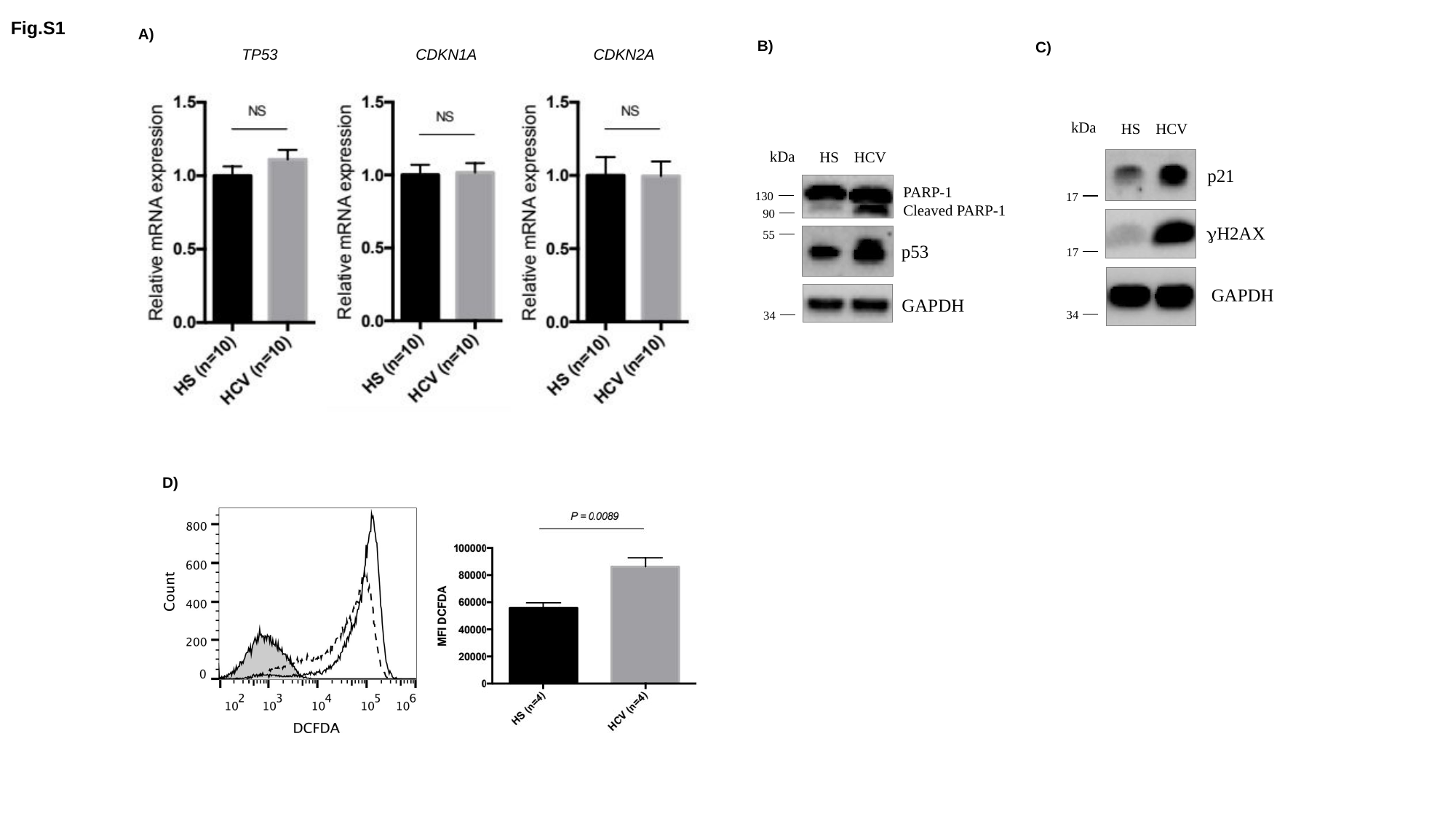

Fig.S1
A)
B)
C)
CDKN1A
TP53
CDKN2A
kDa
 HS HCV
p21
17
gH2AX
17
GAPDH
34
kDa
 HS HCV
PARP-1
Cleaved PARP-1
130
90
55
p53
GAPDH
34
D)
