# Supplementary material for: Inhibition of TRF2 accelerates telomere attrition and DNA damage in naïve CD4 T cells during HCV infection
Source: Cell Death Dis. 2018 Sep 5;9(9):900. doi: 10.1038/s41419-018-0897-y (PMC6125360; doi:10.1038/s41419-018-0897-y)
Supplement: Supplementary file 2 — Supplementary table 1 [file 41419_2018_897_MOESM2_ESM.docx]

| **Table 1: Primers sequences for quantitative RT-PCR** | |
| --- | --- |
| **Target gene** | **Sequence 5’ to 3’** |
| hTERT-F  hTERT-R | CCAAGTTCCTGCACTGGCTGA  TTCCCGATGCTGCCTGACC |
| TERF1-F  TERF1-R | TGCTTTCAGTGGCTCTTCTG  ATGGAACCCAGCAACAAGAC |
| TERF2-F  TERF2-R | GGTACGGGGACTTCAGACAG  CGCGACAGACACTGCATAAC |
| POT1-F  POT-R | TTCCACTAAAGAGCAGGCAA  TGAAGTTCTTTAAGCCCCCA |
| TINF2-F  TINF2-R | CCAGAAAGGGTTCCCCATAC  TTTACCAGCAGGTGAAGCAG |
| TERF2IP-F  TERF2IP-R | TCTTCTTCAGGCAAATCTGGA  CCTCCTCCCAGAAGCTCAA |
| TPP1-F  TPP1-R | TCACCAGATCAGCCACATTC  GGAAAGACTCTCGGAGCTG |
| TP53-F  TP53-R | ATGGAGGAGCCGCAGTCAGAT  GCAGCGCCTCACAACCTCCGTC |
| CDKN1A-F  CDKN1A-R | CGATGGAACTTCGACTTTGTCA  GCACAAGGGTACAAGACAGTG |
| CDKN2A-F  CDKN2A-R | AGACTTGGGTGGAAGAGGA  TAATCATCACAGCTGTTCGG |
| GAPDH-F  GAPDH-R | TGCACCACCAACTGCTTAGC  GGCATGGACTGTGGTCATGAG |
